# Structural brain abnormalities in a single gene disorder associated with epilepsy, language impairment and intellectual disability

**DOI:** 10.1016/j.nicl.2016.07.016

**Published:** 2016-08-04

**Authors:** Joe Bathelt, Duncan Astle, Jessica Barnes, F. Lucy Raymond, Kate Baker

**Affiliations:** a MRC Cognition & Brain Sciences Unit, Cambridge, United Kingdom; b Department of Medical Genetics, Cambridge Institute for Medical Research, University of Cambridge, Cambridge, United Kingdom

**Keywords:** Language, Human genetics, Cognitive development, Cortical morphology, Diffusion-weighted imaging

## Abstract

Childhood speech and language deficits are highly prevalent and are a common feature of neurodevelopmental disorders. However, it is difficult to investigate the underlying causal pathways because many diagnostic groups have a heterogeneous aetiology. Studying disorders with a shared genetic cause and shared cognitive deficits can provide crucial insight into the cellular mechanisms and neural systems that give rise to those impairments. The current study investigated structural brain differences of individuals with mutations in *ZDHHC9*, which is associated with a specific neurodevelopmental phenotype including prominent speech and language impairments and intellectual disability. We used multiple structural neuroimaging methods to characterise neuroanatomy in this group, and observed bilateral reductions in cortical thickness in areas surrounding the temporo-parietal junction, parietal lobule, and inferior frontal lobe, and decreased microstructural integrity of cortical, subcortical-cortical, and interhemispheric white matter projections. These findings are compared to reports for other genetic groups and genetically heterogeneous disorders with a similar presentation. Overlap in the neuroanatomical phenotype suggests a common pathway that particularly affects the development of temporo-parietal and inferior frontal areas, and their connections.

## 1 Introduction

Childhood speech and language problems are highly prevalent, but the neurodevelopmental mechanisms contributing to these impairments are not well understood (Grigorenko, 2009, Newbury and Monaco, 2010, Webster and Shevell, 2004). Developmental speech and language problems typically have a heterogeneous aetiology; this variability means that despite their general prevalence it is difficult to identify the pathways (biochemical, cellular, neural systems) that result in these cognitive deficits. However, it is increasingly possible to identify small groups of individuals who share the same rare genetic cause of developmental language disorder. Although rare, neuroimaging studies of disorders that combine aetiological homogeneity with cognitive specificity offer a unique window into the dysregulation of brain systems relevant to common neurodevelopmental disorders in the general population. For example, the study of white matter organisation in Williams syndrome has highlighted distinct visual and facial processing pathways (Meyer-Lindenberg et al., 2006). Similarly, the study of a rare familial speech disorder (KE family, *FOXP2* mutation) has highlighted the importance of striatal systems and cortico-striatal networks for motor speech control and emergent higher-order language skills (Liégeois et al., 2011, Watkins, 2011).

The current study investigated the structural brain differences of individuals with mutations in *ZDHHC9*, a recurrent cause of X-linked Intellectual Disability (XLID; Raymond et al., 2007). Systematic assessment of clinical history across multiple XLID-associated genes led to the observation that *ZDHHC9* mutations are associated with surprisingly homogeneous neurological and cognitive features (Baker et al., 2015). Specifically, a high proportion of the *ZDHHC9* group had a history of childhood seizures similar to Rolandic Epilepsy (RE, also known as Benign Epilepsy with CentroTemporal Spikes or BECTS). In view of the known association between BECTS and developmental language disorders (Clarke et al., 2007, Datta et al., 2013, Monjauze et al., 2005, Overvliet et al., 2011), we went on to obtain quantitative assessments of both linguistic and non-linguistic abilities applying standardised methods. Carer-report questionnaires highlighted communication abilities on average 15 standardised points lower than motor skills or daily living skills and 20 standardised points lower than socialisation skills (median Vineland Adaptive Behaviour scores: communication 53, daily living skills 73, socialisation 67, motor skills 67). We conducted neuropsychological assessments of oromotor abilities and speech production in cases and IQ-matched controls, rated blind to genetic diagnosis by speech and language therapists. We found that mutations in *ZDHHC9* are associated with persistent deficits in oromotor control, verbal fluency and expressive language, and that speech and expressive language functions were significantly more impaired than in age-matched and IQ-matched individuals with mutations in other XLID genes. Hence despite IQ differences between BECTS and the *ZDHHC9* group, this monogenic disorder is associated with a developmental impairment in communication skills not common to all causes of X-linked ID, and reminiscent of the developmental communication impairments associated with RE.

To date, the neurobiological basis for speech and language deficits in individuals with a history of RE has proven elusive, perhaps because of aetiological heterogeneity and variability in cognitive outcome in this group. Previous investigations of the neural correlates of language deficits in RE identified reduced cortical thickness in perisylvian areas (Overvliet et al., 2013a, Pardoe et al., 2013) and white matter changes in the parietal and temporal lobe (Ciumas et al., 2014, Xiao et al., 2014). The observation of RE-like speech and language difficulties in individuals with *ZDHHC9* mutations provides an opportunity to further specify the neural basis for RE-associated developmental language disorder in a group with defined aetiology.

Preliminary neuro-radiological assessment and volumetric analyses indicated no gross morphological abnormalities in the *ZDHHC9* group other than hypoplasia of the corpus callosum and reduced volume of subcortical structures including the thalamus and striatum (Baker et al., 2015). In the current study, we extended these initial observations by measuring the impact of *ZDHHC9* mutation on brain organisation using MRI focussing on global and regional cortical thickness and surface area, and on white-matter integrity. Furthermore, we used tractography to explore the microstructural integrity of cortical association tracts, regional projections of the corpus callosum and thalamo-cortical radiations, Finally, the integrity of tracts related to language functions (arcuate fasciculus, uncinate fasciculus) was assessed to investigate possible neural correlates of language deficits in this group.

In summary, the current study takes a holistic view of brain development in the *ZDHHC9* group, enabling similarities and differences to published results in groups with a similar cognitive and clinical phenotype (RE, ID, dyspraxia of speech) to be assessed, and to highlight unique features pointing towards molecular and developmental pathways of cognitive outcome.

## 2 Participants & methods

### 2.1 Participants

This study was performed in accordance with the Declaration of Helsinki. The study was approved by the Central Cambridge Research Ethics Committee (REC 11/0330, IRAS 83633). Written informed consent was obtained from adults, or from parents of individuals under the age of 16 years. The study recruited 7 males with inherited loss of function mutations in the *ZDHHC9* gene (Age in years: mean = 29.13, SE = 4.86, Range = 13.83–41.83). Mutation analysis and biochemical characterisation of mutations have been previously reported (Raymond et al., 2007, Mitchell et al., 2014). The *ZDHHC9* group was compared to 7 males individually matched in age ± 2 years (Age in years: median = 23.38, mad = 18.72, Range = 10.17–42.5). Comparison subjects were recruited by local advertisement and had no history of neurological illness or cognitive impairment. Statistical analysis indicated no significant difference in age between the groups (Wilcoxon signed-rank test: W = 40.6, *p* = 0.711).

For detailed description of clinical and cognitive characteristics of the *ZDHHC9* group see Baker et al., 2015. In summary, all individuals with a *ZDHHC9* mutation had mild to moderate intellectual disability (full-scale IQ: median = 64.5, Range = 57–73; verbal IQ median 63.5, performance IQ median 68). 5 individuals had a history of epilepsy, with seizure characteristics and EEG features similar to the Rolandic epilepsy spectrum. At the time of MRI acquisition, 1 participant reported seizures within the previous 3 months, and 3 were currently received anti-epileptic medication (carbemazapine *n* = 1, carbemazapine and lamotrigine *n* = 1, phenytoin *n* = 1). Vineland scores (Sparrow et al., 2005) indicated impaired communication abilities in comparison to other domains of function, with stronger receptive language abilities compared to expressive and written language abilities in the *ZDHHC9* group. The Verbal Motor Production Assessment for Children (VMPAC) (Hayden and Square, 1999) indicated significant oromotor difficulties in the *ZDHHC9* group, including lower performance than IQ-matched controls in tests of speech and non-speech oral control, sequencing, voice characteristics, and connected speech.

### 2.2 MRI acquisition

Magnetic resonance imaging data was acquired at the MRC Cognition and Brain Sciences Unit, Cambridge U.K. All scans were obtained on the Siemens 3 T Tim Trio system (Siemens Healthcare, Erlangen, Germany), using a 32-channel quadrature head coil. The imaging protocol consisted of two sequences: T1-weighted MRI and a diffusion-weighted sequence.

T1-weighted volume scans for surface analysis were acquired using a whole brain coverage 3D Magnetisation Prepared Rapid Acquisition Gradient Echo (MP RAGE) sequence acquired using 1 mm isometric image resolution. Echo time was 2.98 ms, and repetition time was 2250 ms.

Diffusion scans were acquired using echo-planar diffusion-weighted images with an isotropic set of 60 non-collinear directions, using a weighting factor of b = 1000 s mm^− 2^, interleaved with 4 T2-weighted (b = 0) volumes. Whole brain coverage was obtained with 60 contiguous axial slices and isometric image resolution of 2 mm. Echo time was 90 ms and repetition time was 8400 ms.

### 2.3 Cortical morphology analysis

Structural T1-weighted images were analysed with surface-based methods that allow more accurate local mapping of the cortical morphology compared to voxel-based methods. Two commonly used measures reflecting different cellular parameters were derived for the current analysis: cortical thickness and cortical surface area. Broadly speaking, cortical surface area reflects the number of cortical columns, whereas cortical thickness is determined by the number of cells within that column (Raznahan et al., 2011). Other authors suggest that cortical area is tied to the volume of white matter beneath the cortex (Worker et al., 2014). Inter-individual differences in cortical morphology have been linked to age (Schmitt et al., 2014), gender (Sowell et al., 2007), cognitive ability (Schnack et al., 2015), disorders such as intellectual disability and attention deficit hyperactivity (Saute et al., 2014, Zhang et al., 2011), and genetic factors (Joshi et al., 2011, Panizzon et al., 2009, Schmitt et al., 2014, Strike et al., 2015).

For the current analysis, T1-weighted images were processed using the FreeSurfer v5.3.0 (http://surfer.nmr.mgh.harvard.edu/) recon-all pipeline. Detailed description of FreeSurfer algorithms are available from the published literature (Dale et al., 1999, Fischl, 2012, Fischl and Dale, 2000, Fischl et al., 2004). In summary, after correction for magnetic field inhomogeneities, skull stripping and intensity normalisation, surface reconstruction is achieved through segmentation of the boundary between subcortical white matter and grey matter based on intensity differences. Next, a triangular mesh is generated to construct a three dimensional representations of the cortical sheath. Defects in brain mask, GM or WM volumes were manually corrected if necessary and the surface generation steps were repeated on the corrected volumes. All surface reconstructions were visually inspected and incorrect GM/WM segmentation was corrected if necessary following the FreeSurfer guidelines (https://surfer.nmr.mgh.harvard.edu/fswiki/FsTutorial/TopologicalDefect_freeview).

Surface-based registration was used for group level comparison (Fischl et al., 1999). After surface reconstruction, surfaces were co-registered to a spherical atlas, and subsequently parcellated for region-wise comparison (Fischl et al., 2004). For comparison of cortical morphology, cortical thickness was measured in the surface space of each participants as the mean of the two shortest distances between the pial and the white matter mesh (Fischl and Dale, 2000). Surface area was calculated as the sum of the areas of each vertex falling within a given ROI of the cortical parcellation in each subject's native space. The spatial distribution of cortical measures was smoothed using a Gaussian kernel with 10 mm radius.

For statistical comparison, surface maps with morphometric values were projected onto the FreeSurfer average surface (fsaverage5). Normality of surface measures was assessed at each vertex using the Shapiro-Wilk test (Ghasemi and Zahediasl, 2012). Significant deviances from normality were very rare and scattered over the cortex (Percentage of significant deviance from normality: *ZDHHC9*: thickness = 0.446%, area = 0.492%; control: thickness = 1.205%, area = 0.896%). Because the data met normality assumptions, morphometric values were compared using standard pairwise *t*-tests that provide greater statistical power. Probabilities were corrected for multiple comparisons across both hemispheres using false discovery rate correction with the Benjamini-Hochberg algorithm (Hochberg and Benjamini, 1990). These calculations were carried out using in-house software based on the Scientific Tools for Python package (SciPy) v0.17 (Jones et al.).

### 2.4 White matter analysis

Diffusion-weighted imaging allows the quantification of water diffusion in vivo. Based on the diffusion measurement a diffusion model can be fitted to estimate the orientation of maximum diffusion presumed to be co-aligned with the underlying tissue orientation within each voxel. Diffusion-based imaging is the only available method to assess white matter structure in humans in vivo and has provided many insights into the role of white matter structures in health and disease since its inception in the early 1990s (Besseling et al., 2012, Dell'Acqua and Catani, 2012, Johansen-Berg and Behrens, 2006).

In the current study, MRI scans were converted from the native DICOM to compressed NIfTI-1 format using the dcm2nii tool developed at the McCauseland Centre for Neuroimaging ([http://www.mccauslandcenter.sc.edu/mricro/mricron/dcm2nii.html]). Subsequently, the images were submitted to the DiPy v0.8.0 implementation (Garyfallidis et al., 2014) of a non-local means de-noising algorithm (Coupe et al., 2008) to boost signal to noise ratio. Next, a brain mask of the b0 image was created using the brain extraction tool (BET) of the FMRIB Software Library (FSL) v5.0.8. Motion and eddy current correction were applied to the masked images using FSL routines. The corrected images were re-sliced to 1 mm resolution with trilinear interpolation using in-house software based on NiBabel v2.0.0 functions ([http://nipy.org/nibabel/]). Finally, fractional anisotropy maps were created based on a diffusion tensor model fitted through the FSL dtifit algorithm (Behrens et al., 2003, Johansen-Berg et al., 2004). All data processing was carried out on a computer cluster under Scientific Linux release 6.6 (64bit).

### 2.5 Tract-based spatial statistics (TBSS)

For whole-brain comparison, FA maps were analysed using tract-based spatial statistics (TBSS) (Smith et al., 2006), which provides a voxel-by-voxel whole-brain analysis for group comparisons. Initially, FA maps were affine-aligned to the MNI52 standard space. Next, the mean FA image of the whole sample was created and thresholded at an FA value of 0.2 to create a white matter skeleton representing the centre of the tracts common to all images. FA values were then projected onto these skeletons for voxel-wise statistical comparisons using the Threshold-Free Cluster Enhancement method, which adjusts statistics for multiple comparisons across space. Statistical results are reported for group comparisons including mean-centred age as a covariate.

### 2.6 Tractography

Global measures of diffusion parameters based on the diffusion tensor model may be influenced by the definition of the tract skeleton and differences in crossing fibres (Bach et al., 2014). In order to address these short-comings, the integrity of particular white matter pathways was further investigated using tractography based on a model that is better suited to resolve crossing fibres. Tractography is a method used to follow the dominant directions of diffusion within each voxel to reconstruct white matter pathways based on regions of interest (ROI) (Chanraud et al., 2010, Le Bihan, 2003, Wedeen et al., 2005). Eigenvector and FA maps were calculated from the diffusion-weighted images in MRTrix (Tournier et al., 2012). A spherical constrained deconvolution (CSD) model was fitted to the 60 gradient direction images using a maximum harmonic order of 8. Correct anatomical orientation of CSD glyphs was visually inspected for white matter tracts of known orientation (corpus callosum, cortico-spinal tract).

The tractography approach followed the recommendations given for MRTrix software (Tournier et al., 2012): The fibre tracking algorithm was set to a minimum and maximum track length of 10 mm and 200 mm respectively. The minimum radius of curvature was set to 1 mm and the track size to 0.2 mm. The track termination threshold was set to an FA value of 0.1. Definition of region of interest was based on previous reports in the literature. ROIs were defined on FA maps. Overlays of eigenvector maps or co-registered T1-weighted images were used to aid the identification of ROIs. Reconstructions were compared to reference atlases to establish anatomical correspondence (Catani and de Schotten, 2015).

Subsequently, streamlines were propagated probabilistically with a target of 150,000 streamlines using MRTrix functions. Tracts of interest (uncinate fasciculus, corpus callosum, cortico-spinal tract, thalamic radiations) were selected from whole-brain tractography using atlas-based approaches described below. The resulting tracts were exported to TrackVis format for virtual in-vivo dissection (Catani and Thiebaut de Schotten, 2008). ROI delineation for each tract of interest is described below. For volume comparisons, maps of streamline counts were thresholded (> 1 streamline per voxel) and binarised to calculate tract volumes with fslstats.

#### 2.6.1 Corpus callosum

The corpus callosum (cc) was segmented according the scheme by Hofer et al. (Hofer and Frahm, 2006). The cc was identified on a medial sagittal slice and segmented to the proportions in the segmentation scheme using voxel counts. The volume of the corpus callosum was estimated from the voxel counts on a medial slice using MRIcron software (version from the 6th of June 2013).

#### 2.6.2 Thalamic radiations

For reconstruction of connections of the thalamus with cortical regions, streamlines from whole-brain tractography were co-registered to FreeSurfer-processed T1-weighted images using a rigid transform with normalised correlation ratio as a cost function as implemented in FSL FLIRT (Jenkinson and Smith, 2001). Binary masks for the frontal, pre-central, post-central, parietal, temporal, and occipital cortex in the left and right hemisphere were created from automatic parcellation of the cortical white matter surface according to the Desikan-Killany atlas performed using FreeSurfer software (Klein & Tourville, 2012). Thalamus ROIs were defined by hand separately for each hemisphere on an axial slice of the T1-weighted image. Streamlines were selected that traversed both thalamic and target cortical ROIs for comparison of diffusion measures.

#### 2.6.3 Arcuate fasciculus

The arcuate fasciculus was reconstructed using a ROI placed on an axial slice above the body of the corpus callosum. The ROI was identified as a half-moon shaped region lateral to the corona radiata as described by Catani and Thiebaut de Schotten, (2008).

#### 2.6.4 Uncinate fasciculus

The uncinate fasciculus (UF) was reconstructed using the method described by Catani et al. (2008) (Catani and Thiebaut de Schotten, 2008). A two ROI approach with one ROI placed in the temporal lobe on the most posterior coronal slice that showed a clear separation between temporal and frontal lobe. The second ROI was positioned in a high-FA ventral region of the frontal lobe proximal to the temporal lobe.

#### 2.6.5 Cortico-spinal tract

The cortico-spinal tract was reconstructed separately for each hemisphere using a 2 ROI approach. A spherical seed ROI with a diameter of 20 mm was placed in a high-FA region within the cerebral peduncle on the most dorsal level of the pons. A second spherical inclusion ROI with a diameter of 20 mm was placed to include the pre- and post-central gyrus.

### 2.7 Statistical analysis

Diffusion measures for each tract were extracted as the mean across all voxels that contained streamlines for each tract. Because of the limited sample size, median and median deviance were used to describe distributions in the control and *ZDHHC9* case groups. For statistical comparison, the non-parametric Wilcoxon signed rank tests was used for paired samples of *ZDHHC9* cases with control participants of the same age (± 2 years). Bonferroni correction was used to account for multiple comparisons. Statistical analyses were carried out in R v3.1.2 using functions of the ‘stats’ package (The R Development Core Team, 2008).

## 3 Results

### 3.1 Cortical morphology

#### 3.1.1 Global measures of segmentation volumes

A Wilcoxon signed rank test indicated that there was no differences in FreeSurfer-derived intracranial volumes between the *ZDHHC9* and control group (*ZDHHC9*: median = 1,622,699, mad = 54,657; control: median = 1,636,124, mad = 111,567 [all values in mm^3^]; W = 28, *p* = 1). There was also no indication of a significant differences between groups in total grey matter or white matter volume (Total grey matter volume: *ZDHHC9*: median = 69,073, mad = 56,916; control: median = 690,703, mad = 27,631; W = 23, *p* = 0.602; total white matter volume: *ZDHHC9*: median = 439,017, mad = 38,098; control: median = 477,671, mad = 31,706; W = 15, *p* = 0.1473).

#### 3.1.2 Cortical thickness

**Fig. 1:**
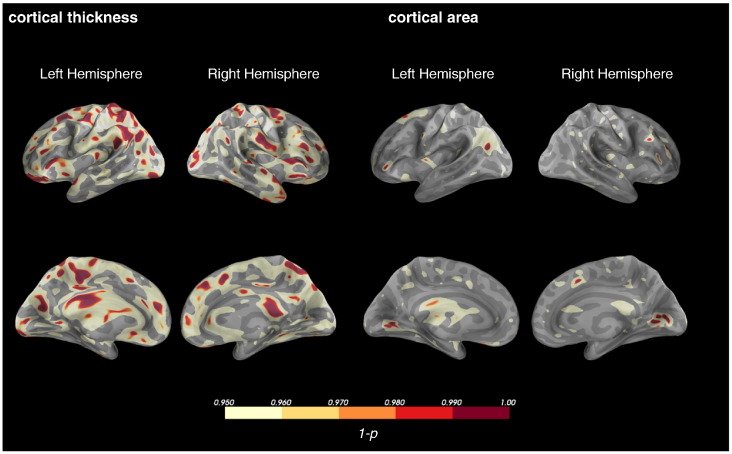
Vertex-wise analysis of cortical volume and thickness comparing the *ZDHCC9* and control group. Statistical analysis was based on a paired *t*-tests and false discovery rate adjustment for multiple comparison including both hemispheres. Decreased cortical thickness was found in the *ZDHHC9* group, particularly in areas surrounding the temporo-parietal junctions and parietal lobule.

Comparison of mean cortical thickness across the entire cortical surface indicated a significant main effect of participant group with lower mean cortical thickness in the *ZDHHC9* group (all values in mm, *ZDHHC9*: median = 2.13, mad = 0.21, 25%ile-75%ile = 1.99–2.27; control: median = 2.53, mad = 0.09, Range = 2.48–2.58, Wilcoxon signed-rank test: W = 49, *p* = 0.002). Follow-up analysis using a general linear model including intracranial volume as a regressor indicated no significant influence of intracranial volume differences on group effects on mean cortical thickness (Effect of intracranial volume: F(1,10) = 0.827, *p* = 0.384). Vertex-wise comparison of cortical thickness across both hemispheres showed reductions in the *ZDHHC9* group, particularly in areas surrounding the temporo-parietal junctions and parietal lobule (see Fig. 1).

#### 3.1.3 Cortical surface area

Comparison of total surface area indicated a significant difference between groups with higher surface area in the *ZDHHC9* group in the left and right hemisphere (all values in m^2^, *ZDHHC9*: median = 0.74, mad = 0.03, 25%ile-75%ile = 0.72–0.75; control: median = 0.71, mad = 0.01, 25%ile-75%ile = 0.69–0.7; W = 2, *p* = 0.005). Vertex-wise comparison of cortical area across the cortical surface in both hemispheres indicated focal increases in the medial occipital lobe bilaterally, the left posterior temporal lobe, and the left inferior frontal lobe in the *ZDHHC9* group (see Fig. 1).

### 3.2 White matter

#### 3.2.1 Whole-brain analysis of diffusion parameters

**Fig. 2:**
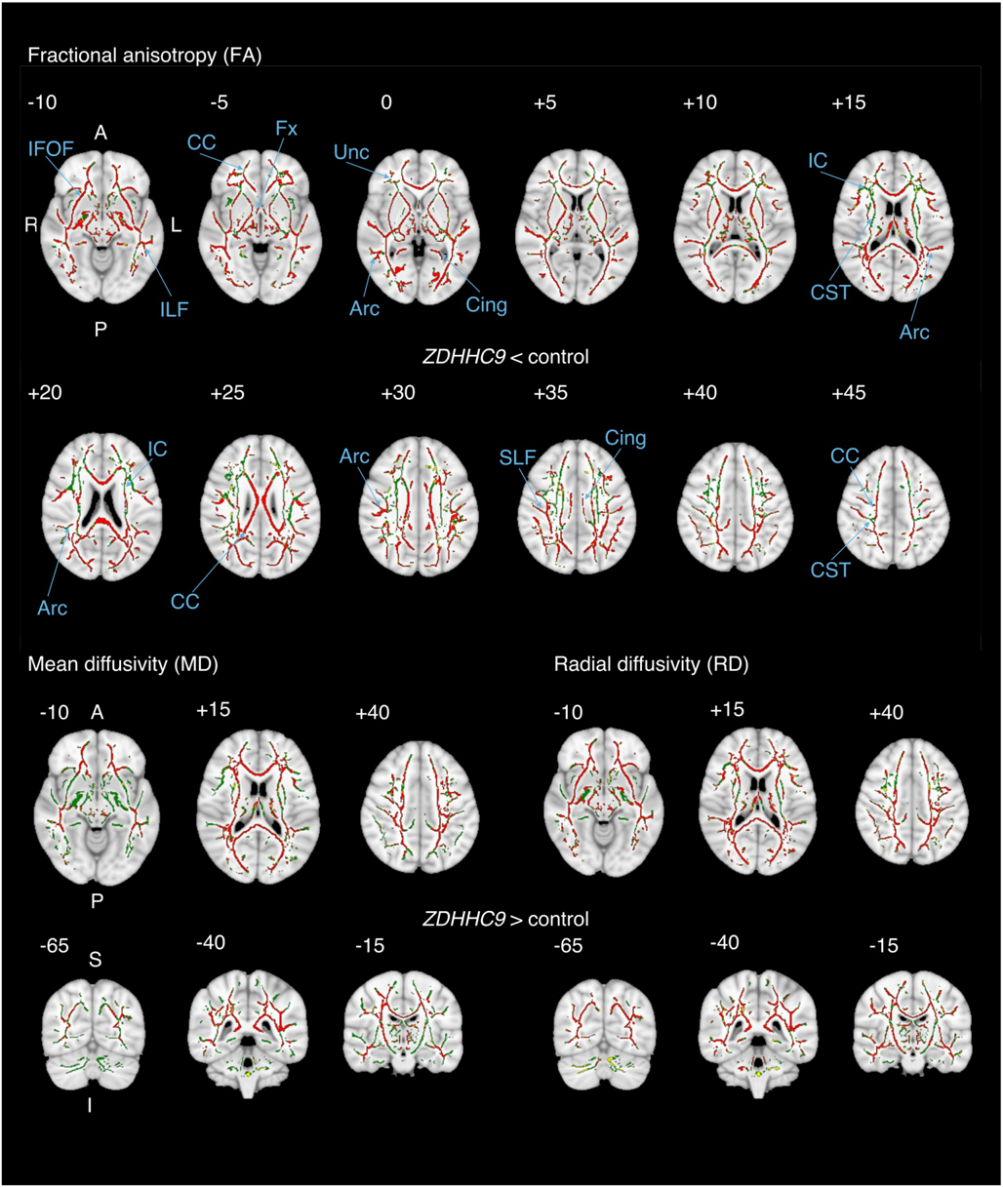
Overview of tract-based spatial statistics (TBSS) results comparing fractional anisotropy (FA) between patients with ZDHHC9 mutation and typical controls adjusted for participant age. Results are presented superimposed on the T1-weighted MNI152 brain at 1 mm^3^ resolution. The top figure shows significant reductions in FA on *p* < 0.05 significance level in the *ZDHHC9* case group compared to the control group are shown in red. The bottom rows show significant increases in MD and RD in the *ZDHHC9* case group. Green lines show the location of the mean FA skeleton. The numbers indicate the axial position with reference to the MNI coordinate system. Annotations highlight key white matter structures. Abbreviations: Arc: arcuate fasciculus, CC: corpus callosum, Cing: cingulate, CST: cortico-spinal tract, IC: internal capsule, ILF: inferior longitudinal fasciculus, Fx: Fornix, SLF: superior longitudinal fasciculus, Unc: Uncinate fasciculus. (For interpretation of the references to colour in this figure legend, the reader is referred to the web version of this article.)

Reductions in fractional anisotropy were found in the *ZDHHC9* group compared to the control group in one very large region (1 cluster of 85,502 voxels at *p* < 0.05 and 39,880 at *p* < 0.001). Peaks within this cluster were observed in the left anterior thalamic radiations, the cerebellar white matter, the body and splenium of the corpus callosum, the left cingulum, bilaterally in the inferior longitudinal fasciculus, and the right inferior fronto-occipital fasciculus (see Table 1). There were no statistically significant increases in FA in the *ZDHHC9* group relative to controls. A similar pattern of increased mean diffusivity (MD) and radial diffusivity (RD) was also found (see Fig. 2 & Table 1).

### 3.2.2 Tractography

#### 3.2.2.1 Corpus callosum

**Fig. 3:**
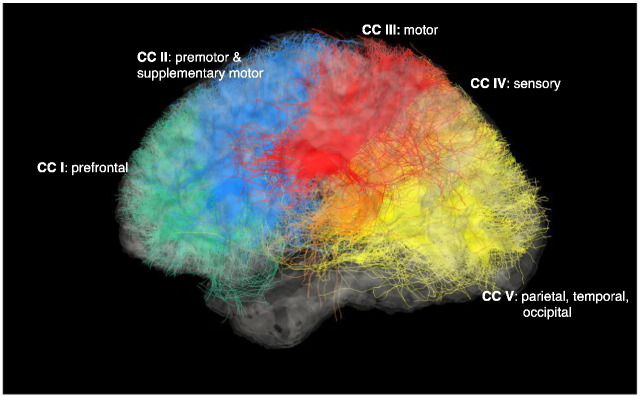
Visualisation of projections of the corpus callosum (cc) in segments in the Hofer and Frahm (2006) parcellation scheme for a control participant. Projections of the anterior segment of the cc mainly contain fibres of the prefrontal cortex. The second segment consists of fibres crossing between premotor and supplementary motor cortex. The third segment holds motor cortex projections. The fourth segment is made up of fibres projecting to sensory areas of the parietal lobe. The most posterior segments contain fibres of the parietal, temporal, and occipital lobe (Hofer and Frahm, 2006).

Statistical comparison indicated significantly reduced volume of projections of the anterior corpus callosum (CI) in the *ZDHHC9* case group compared to controls (W = 1, *p* = 0.023, see Table 2, see Fig. 3 for an illustration of the segmentation). Analysis of FA showed significantly lower FA in the *ZDHHC9* group for all segments of the corpus callosum. Mean diffusivity (MD) was significantly increased in the *ZDHHC9* group in all segments of the corpus callosum (*p* < 0.05), except segment CV (W = 45, *p* = 0.07). Radial diffusivity was also found to be higher in the *ZDHHC9* group in all segments (W = 44, *p* < 0.05) apart from segment CV (*p* = 0.111).

##### 3.2.2.2 Thalamo-cortical projections

**Fig. 4:**
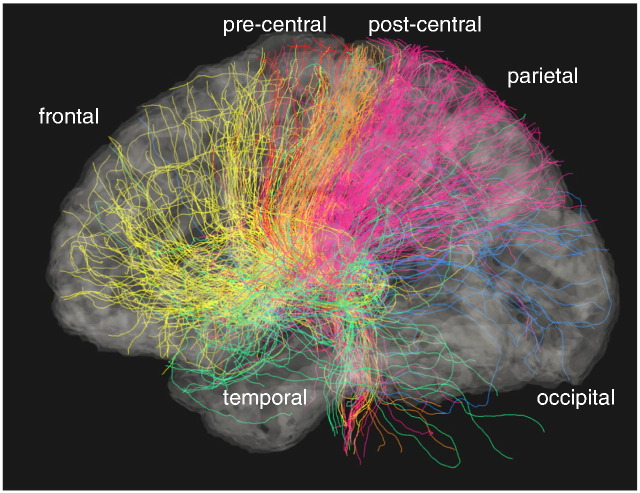
Visualisation of thalamic projections to cortical target areas. Projections from the thalamus to frontal, pre-central, post-central, parietal, temporal, and occipital cortical target areas were distinguished. ROIs were mutually exclusive, i.e. pre-central projections were not included in frontal projections, and post-central projections were not included in the parietal ones.

Statistical comparison of diffusion parameters of projections of the thalamic radiations to cortical target regions indicated significantly lower FA in the *ZDHHC9* case group for projections towards right precentral (W = 38, *p* = 0.012, see Table 3), temporal (W = 38, *p* = 0.029), occipital (W − = 21, *p* < 0.001), and left postcentral cortex (W = 33, *p* = 0.012, see Fig. 4 for an illustration of the projections). Differences in the right precentral and right temporal thalamic radiations were also characterised by significantly higher MD (precentral: W = 170, *p* = 0.003; temporal: W = 170, *p* = 0.03). Significant increases in RD were found for right precentral, right temporal, and right occipital projections (precentral: W = 170, *p* = 0.03; temporal: W = 33, *p* = 0.012; occipital: W = 164, *p* = 0.01). Similar reductions in the left hemisphere that did not survive correction for multiple comparisons.

##### 3.2.2.3 Arcuate fasciculus

**Fig. 5:**
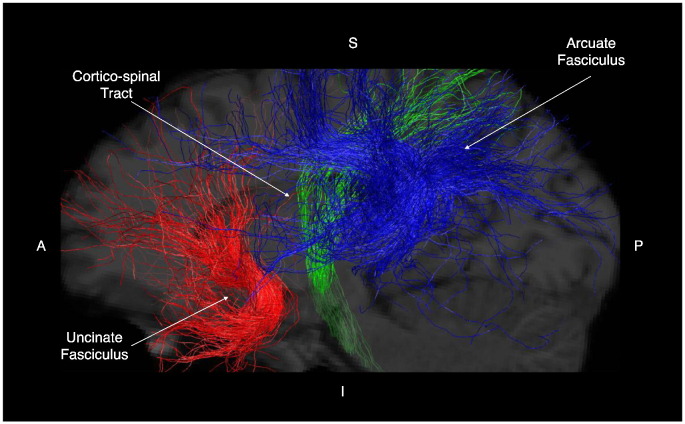
Visualisation of tractography reconstruction of the arcuate fasciculus (AF), cortico-spinal tract (CST), and uncinate fasciculus (UF) in the left hemisphere for a representative participant in the control group.

Statistical analysis indicated no significant difference in volume of the arcuate fasciculus between the *ZDHHC9* and control group (see Table 4 for descriptive statistics and Fig. 5 for an illustration of the tract reconstruction, left Arcuate: W = 5, *p* = 0.066, right Arcuate: W = 6, *p* = 0.105). Comparison of FA values indicated significantly lower FA values in the *ZDHHC9* group (left Arcuate: W = 3, *p* = 0.024, right Arcuate: W = 2, *p* = 0.014). Mean diffusivity (MD) and radial diffusivity (RD) were found to be significantly higher in the *ZDHHC9* group for the right Arcuate (MD: W = 49, *p* = 0.003; RD: W = 49, *p* = 0.003).

##### 3.2.2.4 Uncinate fasciculus

There was no statistically significant difference in volume between the *ZDHHC9* case group and control group for either the left or right Uncinate fasciculus (left Uncinate: W = 25, *p* = 1; right Uncinate: W = 10, *p* = 0.437). FA was found to be significantly reduced in the right Uncinate fasciculus in the *ZDHHC9* case group (W = 4, *p* = 0.042). MD was significantly higher for the left and right Uncinate fasciculus (left Uncinate: W = 45, *p* = 0.045; right Uncinate: W = 45, *p* = 0.042). RD was significantly higher in the left Uncinate (W = 45, *p* = 0.042).

##### 3.2.2.5 Cortico-spinal tract

There was no statistically significant differences in the volume of the cortico-spinal tract between the *ZDHHC9* and control group (see Table 4 for descriptive statistics, F(1,18) = 1.510, *p* = 0.234). FA of the left CST was significantly lower in the *ZDHHC9* case group (W = 0, *p* = 0.003), whilst MD and RD were significantly higher compared to controls (MD: W = 48, *p* = 0.007; RD: W = 49, *p* = 0.003).

### 3.3 Summary of results

In summary, analyses of cortical morphology in individuals with mutations in *ZDHHC9* indicated reductions in cortical thickness. Prominent differences were observed “Rolandic” cortical areas i.e. areas associated with Rolandic-type seizure activity and with language-relevant cognitive functions such as fine control of oral movement, converting articulation, and audio-visual-motor integration (Price, 2010). Global cortical surface area was found to be increased in the *ZDHHC9* group, but vertex-wise comparison indicated no differences between *ZDHHC9* cases and the control group. Collectively these analyses suggest that loss of *ZDHHC9* activity leads to an abnormality of neuronal proliferation, with variation in impact on cytoarchitecture across the cortex.

Total white matter volume did not differ between groups but whole brain analysis of white matter integrity indicated widespread differences in diffusion parameters (lower FA, higher MD and RD). Tractography identified relatively severe reductions in microstructural integrity of anterior projections of the corpus callosum and of thalamo-cortical radiations projecting to precentral, postcentral, temporal and occipital cortex (differences maximal on right side). Analyses of cortical association tracts known to be associated with language competence showed reductions in FA of the arcuate bilaterally and right uncinate fasciculus. FA of the left cortico-spinal tract was also found to be reduced. In summary, examination of white matter in this group suggests that *ZDHHC9* loss of function influences axonal development with impact on cortical, subcortical-cortical and interhemispheric networks.

## 4 Discussion

The current study aimed to comprehensively characterise differences in brain structure associated with a mutation in the *ZDHHC9* gene. Our data demonstrate that *ZDHHC9* mutations are associated with reductions in cortical thickness and white matter microstructural integrity, particularly in regions and networks known to contribute to language function.

Individuals with a *ZDHHC9* mutation showed significantly decreased cortical thickness and increased surface area. Decreased cortical thickness is likely to indicate a reduction of the number or size of cortical cells (Schmitt et al., 2014, Sowell et al., 2007), whereas increased cortical surface area is generally interpreted to reflect atrophy or underdevelopment of white matter beneath the cortex, which leads to deeper sulci (Worker et al., 2014). These results are in line with previous reports of other participant groups with language impairments (see Table 5 for a detailed comparison with published studies on other groups with language deficits). A voxel-based morphometry study of a family with oro-motor deficits associated with mutations in the *FOXP2* gene also indicated reduced grey matter in the pre-supplementary motor cortex and cingulate (Belton et al., 2003, Vargha-Khadem et al., 1998, Watkins et al., 2002). Studies of specific language impairment (SLI) (Soriano-Mas et al., 2009) and language deficits in children with RE (Overvliet et al., 2013a) also identified reduction in areas around the temporo-parietal junction.

Individuals with *ZDHHC9* mutations also show extensive differences in white-matter integrity, in terms of decreased FA and increased mean diffusivity (MD) and radial diffusivity (RD). Mutations in the *CNTNAP2* gene, which are also associated with language deficits, also show reduced FA in the inferior fronto-occipital fasciculus, posterior thalamo-cortical radiations, and uncinate fasciculus (Clemm von Hohenberg et al., 2013, Tan et al., 2010). Likewise, for SLI, increased radial diffusivity of the arcuate fasciculus and reduced FA of the superior longitudinal fasciculus have been reported (Roberts et al., 2014, Verhoeven et al., 2012). In addition, studies of neural differences in RE have also reported widespread reductions in FA, particularly within the corpus callosum, bilateral cingulate gyrus, and left uncinate fasciculus (Gong et al., 2008, Kimiwada et al., 2006).

At the subcortical level, our previous investigation found reduced thalamic volumes in the *ZDHHC9* group (Baker et al., 2015), which is a feature of this genetic group that has not been reported for *FOXP2* mutations, *CNTNAP2* mutations, or for idiopathic groups with similar speech and language difficulties. The current investigation found that FA of posterior thalamo-cortical projections is also reduced in the *ZDHHC9* group. Differences in diffusion properties of the thalamo-cortical radiations have also been reported in temporal lobe epilepsy, a disorder associated with language deficits (Gong et al., 2008, Kimiwada et al., 2006). Previous theoretical accounts have suggested a role of the thalamus in oro-motor control related to speech (Vargha Khadem et al., 2005), but familial speech disorder (*FOXP2*) has hitherto been mostly associated with the caudate nucleus. The current study adds a genetic group with language deficits in combination with intellectual disability that shows effects on the thalamus and thalamo-cortical connections.

In context of studies of structural brain abnormalities in disorders with at least partially overlapping phenotypes (Table 5), our findings suggest convergence in the cortical systems involved in developmental language disorders, irrespective of aetiology. Observed differences in cortical morphology may correlate with immature language processing, rather than being primary causative abnormalities. This proposal is supported by differences in the overt speech phenotypes across these different disorders – *FOXP2* mutation is associated with profound oromotor dyspraxia plus higher order language impairments, whereas oromotor impairments are subtle in Rolandic epilepsy and are not common within the heterogeneous population of individuals diagnosed with specific language impairments of unknown aetiology. The extent of similarity in clinical speech disorder and underlying cognitive impairments between *ZDHHC9*, *FOXP2*, and other monogenic disorders of language development is not yet known and should be the focus of a future comparative study ideally in parallel with longitudinal comparative neuroimaging. In particular, comparison to individuals with *GRIN2A* mutation may be informative in view of association with speech disorders, intellectual disability and focal epilepsy (Turner et al., 2015, Lesca et al., 2013). According to a recent review of neuroimaging studies of language function in adults, these areas are involved in word selection and articulatory planning (inferior frontal lobe) and, covert articulation and audio-visual-motor integration (supramarginal gyrus) (Price, 2010). However, neuroanatomical models based on typical adults or adults with abnormalities arising later in life may not apply in the context of an atypical developmental trajectory, and a within-sample correlative study is required to assess structure-function relationships for the *ZDHHC9* mutation group.

The interpretation of these findings has some important limitations. Because of the rarity of single gene mutations, the possible sample size of studies of this kind is inherently limited. Therefore, the current findings are based on a small sample, which increases the chance of false positive findings and may exaggerate effect sizes (Button et al., 2013). However, investigations of homogeneous aetiology groups as presented in the current work provide unique insight into the effect of single gene disorders that is not afforded in large heterogeneous samples of behaviourally defined groups. In a future study with larger number of participants and more detailed clinical and behavioural evaluations (including, importantly, clinical diagnostic assessments of motor speech disorders) it may be possible to correlate variation in neuroanatomical differences within the *ZDHHC9* group with specific outcomes, to link structural development with functional consequences. At present such correlations are not possible, which also imposes limits on comparison with other disorders (both monogenic and mixed aetiology) where abnormalities of speech and language development have been more highly specified. Another caveat concerns the specificity of the observed effect of *ZDHHC9* on neuroanatomy. Similar to other genetic groups, participants in the current investigation presented with a broader profile of behavioural characteristics, namely overall reductions in IQ. Because IQ reductions are found in all *ZDHHC9* cases but not in the typical control group, IQ differences cannot not be adjusted for statistically, and some of the observed neuroanatomical differences may be non-specific correlates of low cognitive ability rather than reflecting aetiology-relevant or phenotype-relevant pathways. A profile of reduced IQ scores has also been reported for members of the KE family with mutations in the *FOXP2* gene (Vargha-Khadem et al., 1995), but to a lesser extent. It is important to consider that neurodevelopmental disorders do not function like acquired disorders in adulthood. A chronic impairment in a particular domain from birth will have cascading consequences for other systems over the course of development. This is true for groups with genetic disorders like children with mutations in *FOXP2* and *ZDHHC9*; whilst there is a single causal gene, the cognitive and behavioural impairments associated with this mutation will impact upon cognitive development more broadly. Ideally, the impact of *ZDHHC9* mutation on brain structure would be investigated in individuals before, during and after the ages of typical speech and language maturation, however this is not currently feasible. Effects may also appear large in comparison to our control group of typical volunteers. Healthy volunteers for neuroimaging studies are tend to be from a higher socio-economic background, enjoyed more years of education, and perform better on cognitive tests compared to the general population.

To conclude, the overlap in the brain phenotype across many neurodevelopmental language disorders and RE may suggest a common developmental pathway that particularly affects temporo-parietal and inferior frontal areas and their associated networks. Temporo-parietal and frontal cortical regions as well as associated white matter show a particularly prolonged maturation in humans and show large heritability effects (Joshi et al., 2011, Kochunov et al., 2010, Kochunov et al., 2015, Lenroot et al., 2009, Thompson et al., 2001). Previous studies of genetic disorders implicated the regulation of cell migration and cell adhesion as important factors (*CNTNAP2* regulated through *FOXP2*) for the development of these networks (Dityatev et al., 2008, Garcia-Calero et al., 2015). At the cellular level *ZDHHC9* codes for a palmitoylation enzyme, involved in post-translational modification of specific target substrates. Palmitoylation plays an important role in subcellular compartmentalisation and shuttling of proteins between cell compartments (Fukata and Fukata, 2010, Mitchell et al., 2014). For instance, palmitoylation has been found to play an important role in the recruitment of receptors and ion channels at the synapse (El-Husseini et al., 2000, Topinka and Bredt, 1998, Young et al., 2014). The current investigation adds palmitoylation of specific substrates currently unknown as another necessary mechanism for the development of cortical and subcortical networks that mediate language-relevant cognition. A possible pathway may lie in the regulation of the post-synaptic density protein 95 (PSD95), which is implicated in the pathophysiology of both *CNTNAP2* and *DHHC* mutations (Fukata and Fukata, 2010, Rodenas-Cuadrado et al., 2014). Altered regulation of PSD95 and downstream targets along the NRXN–NLGN–SHANK pathway (Bourgeron, 2009, Südhof, 2008) may lead to altered synaptogenesis and imbalance between excitatory and inhibitory activity (Won et al., 2013), with downstream impact on emergent connectivity supporting language development.

Here we provide the first comprehensive characterisation of the structural brain deficits associated with a mutation in *ZDHHC9* – a developmental group with an interesting and homogenous cognitive phenotype. In the coming years the next step will be to explore comparable differences in children with different aetiologies but partially of fully overlapping phenotypes, such that we can draw firm specific conclusions about structure-function relationships.

## Figures and Tables

**Table 1:** Overview of peaks within the cluster of significantly lower FA values in the *ZDHHC9* compared to the control group. Statistical analysis with threshold-free cluster-wise correction for multiple comparison had identified a single cluster. Follow-up analysis provided the highest *t*-values at the coordinates listed above. Anatomical structures were identified through white matter atlas comparison (Catani and de Schotten, 2015).

	MNI coordinates [mm]			*t*-peak
	X	y	z	
Anterior thalamic radiation L	− 16	− 16	18	13.28
Cerebellar white matter	− 4	− 57	− 28	17.59
Body of the corpus callosum	− 8	− 21	26	16.06
Splenium of the corpus callosum	23	− 50	11	13.12
Cingulum L	− 18	− 46	2	13.76
Inferior longitudinal fasciculus L	− 24	− 76	14	12.64
Inferior longitudinal fasciculus R	30	− 65	20	15.45
Inferior fronto-occipital fasciculus R	33	− 62	2	13.72

**Table 2:** Volume and diffusion parameter in corpus callosum (cc) projection segments. Statistical comparison indicated reductions fractional anisotropy (FA) in all segments of the CC in the *ZDHHC9* group compared to controls. Mean diffusivity (MD) and radial diffusivity (RD) were found to be significantly increased in the *ZDDHC9* group. Volume was found to be lower for the anterior segment in the *ZDHHC9* group.

		*ZDHHC9*		Control		W	*p*	*corr-p*	
		Med	Mad	Med	Mad				
Cl	FA	0.28	0.010	0.34	0.015	3	0.004	0.041	*
	MD	1.14	0.144	0.94	0.038	48	0.001	0.012	*
	RD	0.96	0.162	0.74	0.022	48	0.001	0.012	*
	Volume	12.93	11.415	54.89	20.112	1	0.002	0.023	*
CII	FA	0.29	0.044	0.36	0.017	2	0.002	0.023	*
	MD	1.17	0.099	0.93	0.018	49	0.001	0.006	**
	RD	0.97	0.071	0.74	0.012	48	0.001	0.012	*
	Volume	10.76	3.527	37.20	8.475	7	0.026	0.262	
CIII	FA	0.29	0.049	0.36	0.011	2	0.002	0.023	*
	MD	1.20	0.103	0.97	0.030	49	0.001	0.006	**
	RD	1.03	0.062	0.78	0.048	49	0.001	0.006	**
	Volume	4.19	1.358	17.18	10.449	8	0.038	0.379	
CIV	FA	0.28	0.057	0.37	0.020	1	0.001	0.012	*
	MD	1.33	0.171	0.99	0.046	48	0.001	0.012	*
	RD	1.17	0.167	0.80	0.049	48	0.001	0.012	*
	Volume	2.36	2.552	11.58	6.802	10	0.073	0.728	
CV	FA	0.28	0.040	0.37	0.019	3	0.004	0.041	*
	MD	1.31	0.103	1.04	0.087	45	0.007	0.070	
	RD	1.08	0.152	0.83	0.082	44	0.011	0.111	
	Volume	15.21	12.183	49.84	33.480	10	0.073	0.728	

Comparison of diffusion measures within segments of the corpus callosum. Statistical comparison was based on Wilcoxon ranked sign tests corrected for multiple comparison across segments. Abbreviations: fractional anisotropy (FA) [no unit], mean diffusivity (MD) [10^− 3^ ram^2^, s^− 1^], radial diffusivity (RD [10^− 3^ mm^2^ s^− 1^], volume [cm^3^]. (* p < 0.05, ** p < 0.01, *** p < 0.001).

**Table 3:** Values of FA, RD, and MD in the *ZDHHC9* group and the control group. Statistical comparison indicated significantly lower FA for thalamic projections towards the right pre-central, post-central, temporal, and occipital. FA was also significantly lower for left occipital projections.

		*ZDHHC9*		Control		W	*p*	*corr-p*	
		Med	Mad	Med	Mad				
Left frontal	FA	0.27	0.030	0.30	0.013	59	0.077	0.460	
	MD	0.97	0.040	0.98	0.049	123	0.265	1.000	
	RD	0.83	0.048	0.84	0.048	123	0.265	1.000	
Right frontal	FA	0.29	0.037	0.31	0.006	62	0.104	0.621	
	MD	1.00	0.061	0.97	0.057	142	0.044	0.266	
	RD	0.86	0.064	0.81	0.068	140	0.056	0.334	
Left precentral	FA	0.31	0.037	0.35	0.048	60	0.085	0.510	
	MD	0.95	0.039	0.92	0.039	151	0.014	0.085	
	RD	0.80	0.056	0.76	0.053	153	0.011	0.064	
Right precentral	FA	0.33	0.053	0.34	0.015	38	0.005	0.029	*
	MD	1.00	0.056	0.97	0.088	170	0.001	0.003	**
	RD	0.84	0.032	0.81	0.071	170	0.001	0.003	**
Left postcentral	FA	0.29	0.039	0.33	0.032	33	0.002	0.012	*
	MD	0.95	0.083	0.91	0.035	152	0.012	0.073	
	RD	0.82	0.169	0.75	0.023	154	0.009	0.055	
Right postcentral	FA	0.32	0.034	0.35	0.007	21	0.000	0.001	***
	MD	1.03	0.057	0.97	0.027	154	0.009	0.055	
	RD	0.87	0.027	0.82	0.014	164	0.002	0.010	*
Left parietal	FA	0.31	0.051	0.33	0.024	59	0.077	0.460	
	MD	1.00	0.075	0.92	0.053	123	0.265	1.000	
	RD	0.85	0.104	0.75	0.041	123	0.265	1.589	
Right parietal	FA	0.30	0.026	0.35	0.007	62	0.104	0.621	
	MD	1.06	0.057	0.95	0.039	142	0.044	0.266	
	RD	0.89	0.039	0.77	0.048	140	0.056	0.334	
Left temporal	FA	0.27	0.034	0.29	0.014	60	0.085	0.510	
	MD	1.08	0.115	1.03	0.019	151	0.014	0.085	
	RD	0.96	0.117	0.88	0.019	153	0.011	0.064	
Right temporal	FA	0.25	0.038	0.30	0.021	38	0.005	0.029	*
	MD	1.17	0.097	1.07	0.123	170	0.001	0.003	**
	RD	1.01	0.086	0.92	0.113	170	0.001	0.003	**
Left occipital	FA	0.27	0.048	0.29	0.021	33	0.002	0.012	*
	MD	1.07	0.121	0.98	0.026	152	0.012	0.073	
	RD	0.94	0.137	0.82	0.044	154	0.009	0.055	
Right occipital	FA	0.23	0.027	0.31	0.017	21	0.000	0.001	***
	MD	1.16	0.105	1.06	0.053	154	0.009	0.055	
	RD	1.02	0.112	0.89	0.060	164	0.002	0.010	*

Comparison of diffusion measures within the thalamic radiations. Statistical comparison was based on Wilcoxon ranked sign tests corrected for multiple comparison across radiation segments. Abbreviations: fractional anisotropy (FA) [no unit], mean diffusivity (MD) [10^− 3^ mm^2^ s^− 1^], radial diffusivity (RD [10^− 3^ mra^2^ s^− 1^], volume [cm^3^]. (* *p* < 0.05, ** *p* < 0.01, *** *p*<0.001).

**Table 4:** Descriptive statistics for volume, FA, MD, and RD in the arcuate, fasciculus, uncinate fasciculus, and cortico-spinal tract (CST) in the left and right hemisphere.

		*ZDHHC9*		Control		W	*p*	*corr-p*	
		Med	Mad	Med	Mad				
Left arcuate	FA	0.23	0.013	0.28	0.011	3	0.004	0.024	*
	MD	0.99	0.065	0.84	0.039	41	0.038	0.227	
	RD	0.87	0.084	0.72	0.039	44	0.011	0.066	
	Volume	4.73	4.012	9.01	2.979	5	0.011	0.066	
Right arcuate	FA	0.22	0.035	0.29	0.038	2	0.002	0.014	*
	MD	1.02	0.088	0.83	0.036	49	0.001	0.003	**
	RD	0.90	0.065	0.71	0.050	49	0.001	0.003	**
	Volume	5.15	1.327	14.66	2.595	6	0.017	0.105	
Left uncinate	FA	0.22	0.009	0.28	0.035	11	0.097	0.584	
	MD	0.97	0.035	0.89	0.062	47	0.002	0.014	*
	RD	0.86	0.048	0.77	0.086	45	0.007	0.042	*
	Volume	0.08	0.055	2.09	0.464	25	1.000	1.000	
Right uncinate	FA	0.21	0.024	0.31	0.022	4	0.007	0.042	*
	MD	0.97	0.045	0.87	0.010	45	0.007	0.042	*
	RD	0.83	0.011	0.72	0.010	43	0.021	0.128	
	Volume	0.31	0.322	1.88	0.882	10	0.073	0.437	
Left CST	FA	0.38	0.040	0.41	0.014	0	0.001	0.003	**
	MD	0.97	0.057	0.87	0.033	48	0.001	0.007	**
	RD	0.76	0.027	0.67	0.024	49	0.001	0.003	**
	Volume	3.09	2.559	2.11	0.624	6	0.035	0.210	
Right CST	FA	0.42	0.031	0.43	0.040	16	0.318	1.000	
	MD	0.91	0.037	0.86	0.023	40	0.053	0.318	
	RD	0.70	0.039	0.66	0.057	39	0.073	0.437	
	Volume	4.83	3.747	5.50	1.696	23	0.902	1.000	

Descriptive statistics for tractography of the arcuate fasciculus, uncinate fasciculus, and corticospinal tract in the left and right hemisphere. Statistical comparison was based on Wilcoxon ranked sign tests corrected for multiple comparison across segments. Abbreviations: fractional anisotropy (FA) [no unit], mean diffusivity (MD) [10^− 3^ mm^2^ s^− 1^], radial diffusivity (RD [10^− 3^ mm^2^ s^− 1^], volume [cm^3^]. (* *p* < 0.05, ** *p* < 0.01, *** *p* < 0.001).

**Table 5:** Overview of findings in the current investigation compared to published results for other monogenic disorders associated with developmental speech and language impairments, idiopathic Rolandic epilepsy, or idiopathic developmental speech and language impairments. Legend: ↑ increase, ↓ reduction, FA: fractional anisotropy, GM: grey matter, WM: white matter, N/A: not available.

		Genetic disorders associated with language deficits			Heterogeneous diagnostic groups with language deficits		
		ZDHHC9	FOXP_2_	CNTNAP_2_	Rolandic epilepsy	Speech disorder	Specific language impairment
**Volumetric findings**	Cortical	None reported	↓ GM in left pre-supplementary motor area, cingulate cortex, Broca's area	↓ GM bilat. fusiform gyri, post, occipital cortices, right frontal pole	N/A	†GM in bilateral superior temporal gyrus	↑ volume & GM of right perisylvian regions, ↓ volume & GM of left perisylvian regions
	Subcortical	↓ GM bilat. in thalamus, bilat. in caudate	↓ GM bilat. in globus pallidus and putamen ↓ GM bilat. in caudate nucleus	None reported	N/A	None reported	↓ caudate volume
**Cortical morphology**	Cortical thickness	↓ bil. in supramarginal gyrus, superior parietal lobule, inferior frontal gyrus, cingulate cortex	N/A	N/A	↓ bil. areas of frontal and temporal lobe, parietal lobe, ↓ supramarginal gyrus, banks of the superior temporal sulcus, lower	↓ left supra marginal gyrus in children (3–6 y)	N/A
	Cortical surface area	↑ bilateral medial occipital lobe, posterior temporal lobe	N/A	N/A	N/A	N/A	N/A
**White matter findings**		Hypoplasia of the corpus callosum, ↓ FA, ↑ MD, ↑ RD cortical, cortical-subcortical, interhemispheric projections	N/A	↓ FA inferior fronto-occipital fasciculus, bil. post, corticothalamic radiations, uncinate fasciculus	↓ FA ipsilateral to epilepsy focus in corpus callosum, forceps minor; seizure frequency correlated with ↓ FA in corpus callosum, bil. cingulate and left uncinate	↑ WM of corpus callosum and WM of the right lateral occipital cortex	↑ RD of arcuate fasciculus, ↓ FA in superior longitudinal fasciculus
**References**		Baker et al. (2015), present paper	Belton et al. (2003), Vargha-Khadem et al. (1998), Watkins et al. (2002)	Clemm von Hohenberg et al. (2013), Tan et al. (2010)	Ciumas et al., 2014, Kim et al., 2014, Overvliet et al., 2013b, Pardoe et al. (2013), Xiao et al. (2014)	Kadis et al., 2014, Preston et al., 2014	Badcock et al., 2012, Gauger et al., 1997, Girbau-Massana et al., 2014, Leonard et al., 2002, Roberts et al. (2014), Soriano-Mas et al. (2009), Verhoeven et al. (2012)

## Appendix A Supplementary data

Supplementary analysis of tract volume at different streamline density thresholds. Image 1

3D neuroimaging data showing the comparison between the *ZDHCC9* and control group. The values indicate 1-p for lower FA and higher MD and RD in the *ZDHHC9* group corrected for multiple comparison using threshold-free cluster enhancement. Image 2
