# Supplementary material for: Structural brain abnormalities in a single gene disorder associated with epilepsy, language impairment and intellectual disability
Source: Neuroimage Clin. 2016 Aug 4;12:655–65. doi: 10.1016/j.nicl.2016.07.016 (PMC5053034; doi:10.1016/j.nicl.2016.07.016)
Supplement: Supplementary file 1 — Supplementary analysis of tract volume at different streamline density thresholds. [file mmc1.docx]

**Supplementary Materials**

**Threshold-dependence of tract volumes**

Volume measurements were obtained from streamline tractography by thresholding and binarizing a streamline density image. This necessitates to set a threshold for the minimum number of streamlines that need to pass through a voxel to be included in the tract mask image. In this supplementary analyses, we assessed how robust the results of tract volume comparisons between the groups are to different streamline-per-voxel thresholds.

To this end, we created streamline density maps for each tract. Density maps were then thresholded to only contain voxels above a threshold ranging from above 1 to above 9 streamlines per voxel. The thresholded maps were binarised and the group average total number of voxels was compared between *ZDHHC9* cases and controls using Wilcoxon rank sum tests. Streamline density images were created using DiPy v0.9.2 (Garyfallidis et al., 2014) and Nibabel v2.1.0 (<http://nipy.org/nibabel/#id1>, last visited: 6/7/17). Volumes were calculated using fslstats.

The results indicate that the comparison does vary depending on the choice of the streamline per voxel threshold. However, the variation was small and does not affect the conclusion about significantly differences in tract volume between the groups (see Figure 1).


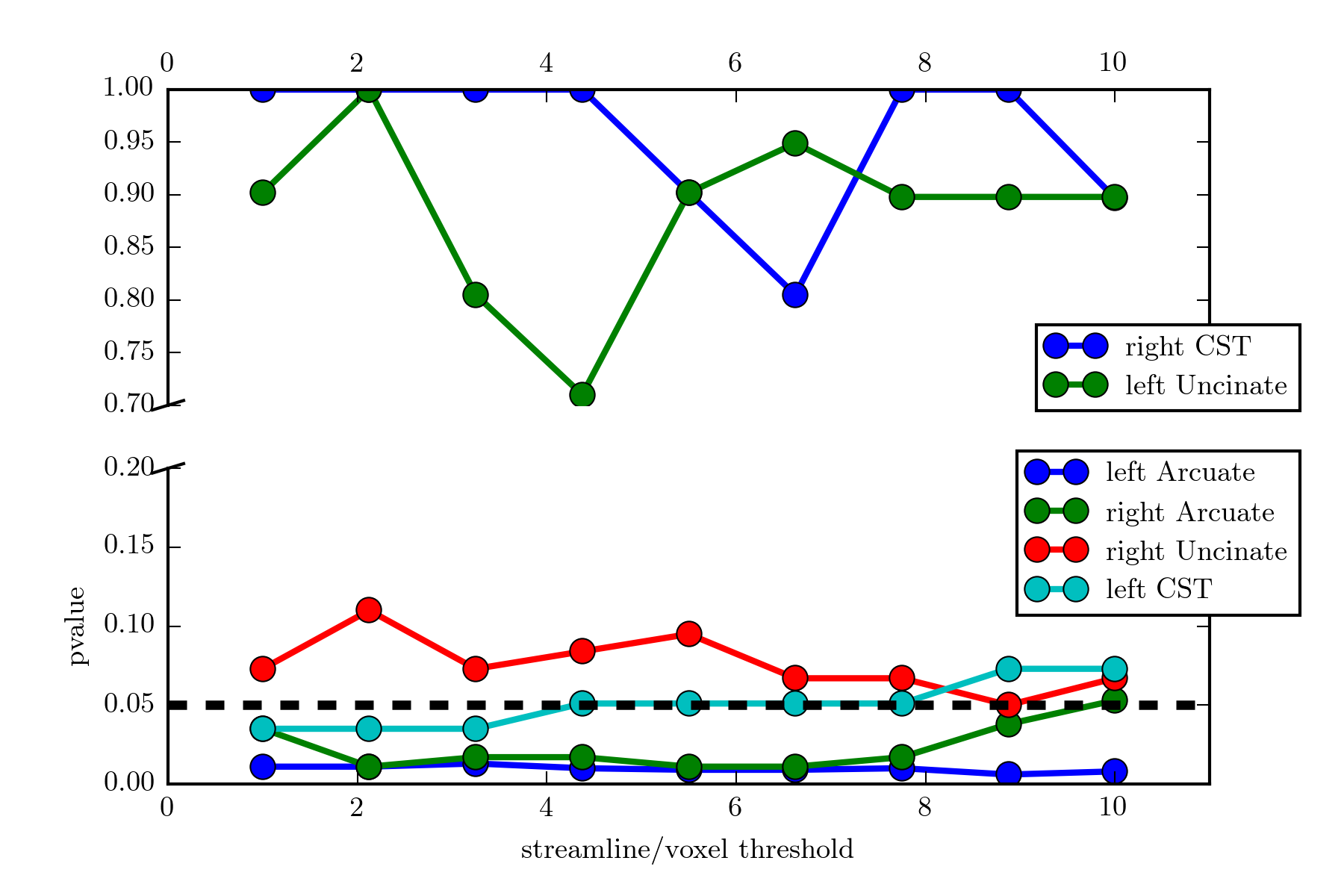


Figure 1: Comparison of tract volume after thresholding at different streamline per voxel cut-offs. The y-axis shows the p-value of a comparison between ZDHHC9 cases and controls using a Wilcoxon rank sum test. The y-axis is discontinuous for better visualisation, but was continuous for the analysis.

**References**

Baker, K., Astle, D. E., Scerif, G., Barnes, J., Smith, J., Moffat, G., et al. (2015). Epilepsy, cognitive deficits and neuroanatomy in males with ZDHHC9 mutations. *Annals of Clinical and Translational Neurology*, *2*(5), 559–569. http://doi.org/10.1002/acn3.196

Garyfallidis, E., Brett, M., Amirbekian, B., Rokem, A., Van Der Walt, S., Descoteaux, M., et al. (2014). Dipy, a library for the analysis of diffusion MRI data. *Frontiers in Neuroinformatics*, *8*(175), 8. http://doi.org/10.3389/fninf.2014.00008
